# Opinion: What do rescue experiments with heterologous proteins tell us and what not?

**DOI:** 10.1007/s00436-021-07247-z

**Published:** 2021-08-05

**Authors:** Adrian Streit

**Affiliations:** Department of Integrative Evolutionary Biology, Max Planck Institute for Developmental Biology, Max-Planck-Ring 9, 72076 Tübingen, Germany

**Keywords:** Non-model organism, Rescue experiment, Biochemical property, Gene function

## Abstract

The recent progress in sequencing technology allowed the compilation of gene lists for a large number of organisms, though many of these organisms are hardly experimentally tractable when compared with well-established model organisms. One popular approach to further characterize genes identified in a poorly tractable organism is to express these genes in a model organism, and then ask what the protein does in this system or if the gene is capable of replacing the homologous endogenous one when the latter is mutated. While this is a valid approach for certain questions, I argue that the results of such experiments are frequently wrongly interpreted. If, for example, a gene from a parasitic nematode is capable of replacing its homologous gene in the model nematode *Caenorhabditis elegans*, it is often concluded that the gene is most likely involved in the same biological process in its own organism as the *C. elegans* gene is in *C. elegans*. This conclusion is not valid. All this experiment tells us is that the chemical properties of the parasite protein are similar enough to the ones of the *C. elegans* protein that it can perform the function of the *C. elegans* protein in *C. elegans*. Here I discuss this misconception and illustrate it using the analog of similar electric switches (components) controlling various devices (processes).

## Introduction

The recent rapid progress in sequencing technology allows the acquisition of an enormous amount of sequence data quickly and relatively inexpensively. As a result, we are approaching complete gene lists for a rapidly increasing number of organisms including many parasitic helminths (International Helminth Genomes Consortium 2019; https://parasite.wormbase.org/index.html; Howe et al. 2017). However, sequence alone provides only very limited information about the function and the interactions of all these genes. Many of these genes are in organisms that are not experimentally tractable when compared with well-established model organisms. One increasingly popular approach to elucidate the functions of genes identified in poorly tractable organisms is to express these genes in a model organism, and then ask what the corresponding protein does in this system when expressed in addition to the corresponding endogenous gene or if the gene is capable of replacing the homologous endogenous one when the latter is mutated. If a gene is capable of replacing its homolog in the model species, it is sometimes concluded that the gene is most likely involved in the same biological process in its own organism because it can perform this function in the model organism. Here, I illustrate why this conclusion is not valid and I evaluate what these experiments actually tell us about the non-model organism’s gene and the function of this gene, and what is lacking. While this critique is not novel, after encountering an increasing number of these experiments while reviewing manuscripts, I decided to explain the pitfalls of these assumptions while illustrating with an analog of electric switches controlling various devices. I deliberately did not include references for specific examples of appropriate or inappropriate interpretations of heterologous expression experiments, as these papers are frequently otherwise well conducted and I have no intention of pointing fingers.

As an example, let’s look at the thoroughly investigated development of specialized third stage larvae in nematodes. Many non-parasitic nematodes, among them the model species *Caenorhabditis elegans* and *Pristionchus pacificus*, can form two alternative third stage larvae, fast developing L3s, which molt into the fourth larval stage after a few hours, and dauer larvae, which can arrest development for up to several months (Androwski et al. 2017; Hu 2007; Karp 2018; Mayer and Sommer 2011). Integrating multiple environmental cues, like population density and food availability, the individual worm switches between the two alternative developmental routes. This developmental switch in *C. elegans* is one of the most intensely studied processes in all of biology (Androwski et al. 2017; Hu 2007; Karp 2018). In *C. elegans*, this complex process at the molecular level involves at least four different cell-signaling cascades (cyclic guanosine monophosphate [cGMP], insulin/IGF-1-like [IIS], transforming growth factor β [TGFβ], and DAF-12 nuclear hormone receptor [NHR] signaling). Many of the mutations that affect dauer formation in *C. elegans* (Dauer defective [Daf] mutations) are in genes for components of these signaling cascades and cause the inactivation or constitutive activation of these pathways. Daf mutations have either a Dauer defective (Daf-d) or a Dauer constitutive (Daf-c) phenotype. Infective third stage larvae (iL3) of some parasitic nematodes are well accepted to be the equivalent of dauer larvae (Androwski et al. 2017; Crook 2014). It is straightforward to identify, based on sequence, genes in a parasitic nematode that appear to be orthologs of *C. elegans* genes involved in the control of dauer formation. These genes can then be expressed in *C. elegans* strains carrying a mutation in the respective endogenous gene and are therefore Daf. If the heterologous (parasite-derived) gene is capable of rescuing the mutation, one may be tempted to conclude that, since it is capable of acting in the control of dauer formation, this gene is most likely involved in the homologous biological process in its natural environment as well, which is the formation of the iL3. However, this conclusion is not valid. Why?

## Mechanic analog

**Fig. 1:**
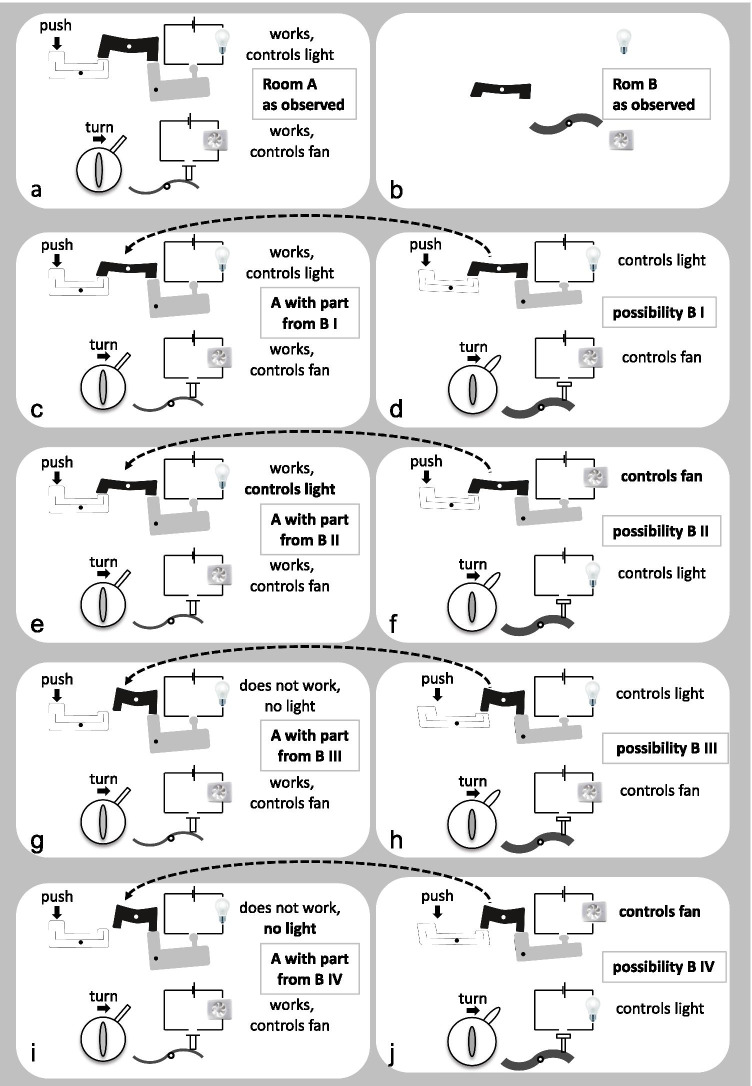
Schematic representation of the switches and electric devices in rooms A and B. (**a**) Room A as observed, corresponding to the well-characterized model organism; (**b**) room B as observed, corresponding to the poorly tractable parasite; (c, e, g, i) the outcomes of the parts exchange experiment if the part was taken from the room B depicted next to it; (d, f, h, j) the four possible situations in room B (possibility B I–IV); (**c**, **d**) possibility B I: the push button switch in room B controls the light (as in room A) and the black part does fit into the switch in room A; (**e**, **f**) possibility B II: the push button switch in room B controls the fan (different from room A) and the black part does fit into the switch in room A; (**g**, **h**) possibility B III: the push button switch in room B controls the light (as in room A) but the black part does not fit into the switch in room A; (**i**, **j**) possibility B IV: the push button switch in room B controls the fan (different from room A) and the black part does not fit into the switch in room A. Notice that there are four different possibilities for room B (**d**, **f**, **h**, **j**) but only two possible outcomes of the replacement experiments, namely the hybrid push button switch works (c = e) or does not work (g = i) and this outcome is independent of which switch controls which device in room B

Genetic regulatory modules can be viewed as switches consisting of multiple components. Each gene represents the manufacturing instructions for one particular part of the switch. I will illustrate my point using an analogy: electric switches, each consisting of three mechanically interacting parts. Imagine a room (room A) with two electric switches, a push button switch and a turning switch, both from manufacturer 1 (schematically represented in Fig. 1a). You know this room well. The push button switch controls the light and is operated by whoever uses the room according to need. The turning switch controls the ventilation and is normally operated by the patrolling night guard in the morning and in the evening. Since you are interested in electrical switches, you also know how the two switches work mechanically. Room A and its conditions correspond to the well-studied model organism *C. elegans*. Now you are in a new, different room (room B) also with a ventilation system and a light. Since there is repair work going on in this room, the power is cut and the switches are disassembled, such that you cannot try them out. However, you manage to get your hands at a few spare parts. Because they look similar to certain parts of the switches at home, you suspect that they are also components of a push button and a turning switch, however, from a manufacturer 2 (Fig. 1b). This corresponds to the poorly tractable parasitic nematode, from which you managed to isolate genes that look similar to well-known *C. elegans* genes.

Now you try to replace the parts in the switches in room A with the parts you brought from room B. If you can successfully replace the black part of the push button switch in room A with the corresponding part from room B (Fig. 1c), you might be tempted to conclude that the latter is most likely also part of a switch that controls the light in room B, as illustrated in Fig. 1d (possibility B I). But now, let’s assume that the situation in room B is as depicted in Fig. 1f (possibility B II). The switches are the same ones as before but now the push button switch controls the fan instead of the light. The outcome of the corresponding exchange experiment is shown in Fig. 1e. Notice that, although Fig. 1d (possibility B I) and f (possibility B II) are different, the outcomes of the replacement experiments (Fig. 1c and e) are the same such that in both cases the part derived from room B is now part of a functional hybrid switch, which controls the light in room A. Therefore, the replacement experiments do not differentiate the origins, B I (Fig. 1d) or B II (Fig. 1f). Similarly, if the exchange experiment does not lead to a functional switch in room A (Fig. 1g and i), the result is the same if the push-button switch the exchanged part was taken from controls the light (possibility B III, Fig. 1h) or the fan (possibility B IV, Fig. 1j) in room B. In both cases, room A remains without a switchable light. Hence, the outcome of the exchange experiment depends only on the shape of the black part in the switch in room B and its capacity (or lack thereof) to connect the white and the grey parts in the switch in room A, but is independent of the device the switch it was taken from controls in room B. If the black part is capable of connecting the two neighboring parts by manufacturer 1 and therefore contributes to a functional hybrid switch, this hybrid switch, because it is in room A, will always control the light and never the fan. If the black part from manufacturer 2 is too short, it fails to bridge the two neighboring components of the switch from manufacturer 1, resulting in a non-functional hybrid switch and no light in room A. In other words, if the replacement experiment is successful, you know that manufacturer 2 builds this particular part similar enough to manufacturer 1 that it can replace the part from manufacturer 1 (in the context of a switch by manufacturer 1). If the part from room B cannot replace the corresponding one in room A, ignoring for the moment the caveats of negative results, this means that the two manufacturers build this particular part differently, such that they are not interchangeable. However, this experiment does not provide any information about what device the corresponding switch controls in room B and who operates it. It is also important to notice that parts can, overall, look rather different and still be able to replace each other provided a few key properties are conserved. Alternatively, two parts may look very similar but not fit because of one crucial difference.

## Back to biology

Obviously, biological systems are much more complex than mechanical switches. The comparison with a cascade of mechanical interactions as outlined in Fig. 1 works best and is most intuitive for signaling cascades where different macromolecules interact with each other physically. Indeed, many mutations isolated in model organisms affect components of a group of phylogenetically widespread signaling pathways, which are used at multiple places and times during the ontogeny of animals (Pires-DaSilva and Sommer 2003). The proteins that make up the core of signaling pathways and their direct interactions tend to be rather highly conserved among different taxa; while what operates these biological switches (the users) and the processes (devices) they control and the regulatory logic according to which the different pathways interact are much more evolutionarily variable, a phenomenon known as “developmental systems drift” (Ewe et al. 2020; Haag et al. 2018; Pires-DaSilva and Sommer 2003; Sommer and Bumbarger 2012). One of the best studied examples among nematodes is the induction of the vulva, the egg-laying and copulatory organ, in the model nematodes *C. elegans* and *P. pacificus*. In both species, the same three cells give rise to the vulva and receive an inductive signal from essentially the same sender. However, while in *C. elegans*, the inductive signal occurs primarily through TGF, *P. pacificus* uses Wnt signaling for the same purpose (Sternberg 2005; Tian et al. 2008; Wang and Sommer 2011; Zheng et al. 2005; reviewed in Haag et al. 2018; Sommer 2012; Sommer and Bumbarger 2012). From this, it appears clear that if *P. pacificus* TGF pathway homologs are capable of replacing their homologous counterparts in *C. elegans* (to my knowledge, no such experiments have actually been reported), in this species, upon ectopic expression, they are expected to act in vulva induction. But, to conclude that they do the same in their species of origin, *P. pacificus*, would be wrong. A number of other examples for developmental systems drift exist in nematodes, for example, in the gene regulatory networks that control endoderm formation or sex determination, and some of them can be observed even between closely related species within single genera (reviewed in Ewe et al. 2020; Haag et al. 2018; Sommer and Bumbarger 2012). This illustrates that the phenomenon is by no means restricted to the comparison of rather distantly related parasitic and free-living species but occurs also between close relatives with very similar live styles. In the dauer/iL3 example mentioned above, developmental systems drift has also been observed. The parasitic nematodes *Strongyloides* spp. have homologous genes for all the signaling pathways involved in dauer formation in *C. elegans* (Hunt et al. 2016; Stoltzfus et al. 2012), or, to stay with our mechanical analog, they have very similar components and switches. The progeny of parasitic *Strongyloides* spp. can either develop into iL3 or into fast-developing non-infective L3s, which give rise to free-living adults (Streit 2017). The iL3 in these parasites and the dauer larvae in *C. elegans* are well accepted to be homologous stages (Crook 2014; Ogawa et al. 2009; Streit 2014; Wang et al. 2009). But, while parts of the genetic regulatory machinery that controls dauer/iL3 development in these two taxa are clearly conserved (Castelletto et al. 2009; Crook 2014; Dulovic and Streit 2019; Ogawa et al. 2009; Stoltzfus et al. 2014, 2012; Wang et al. 2009), other aspects differ. For example, the role for TGF β signaling in *S. stercoralis* iL3 activation seems opposite to its role in *C. elegans* and the epistatic relationship of the IIS and the NHR pathways appears reversed in *S. stercoralis*, compared with *C. elegans* (Stoltzfus et al. 2014, 2012). Again, if an *S. stercoralis* homolog of any gene in one of these pathways is capable of replacing its *C. elegans* homolog in *C. elegans*, it will contribute to a regulatory cascade (switch) that acts in whatever way this pathway functions in *C. elegans*. Such a *C. elegans* strain, with a parasite homolog replacing, or being present in addition to, the endogenous protein may be an excellent tool for studying properties of the parasite protein, for example, screening for or characterizing directly interacting inhibitors. But, it does not provide any conclusive information about whether or not the function (the device it controls) of the pathway it acts in is conserved between *C. elegans* and *S. stercoralis*.

## Conclusion

While the model in Fig. 1 probably most intuitively applies for signaling cascades, it is in principle also valid for other regulatory mechanisms. It is likely that the evolutionary constraints that maintain a component of a regulatory cascade rather constant, and with this frequently capable of replacing the homologous protein in a different organism, are mainly caused by the necessity to interact properly with the immediately adjacent components rather than the biological process it ultimately helps control. Or, in the terms of the analog in Fig. 1, the immediate function of the black part, which is under strong purifying (conserving) selection, is to connect the white and the grey parts. The parts exchange experiment will tell us if the part from room B is capable of fulfilling this function in room A, but not if the switch in room B controls the light or the fan. So, in a rescue or an ectopic expression experiment, if a parasite gene is capable of assuming the function of a *C. elegans* gene in *C. elegans*, it can be concluded that the crucial biochemical and biophysical properties of the parasite protein are similar enough to the ones of the corresponding *C. elegans* protein that it can contribute to a functional module in an otherwise *C. elegans* environment. But, this result is uninformative about the parasite’s biological process the corresponding module is involved in.

## Data Availability

N/A

## Code availability

N/A
